# Supplementary material for: Racial disparities in the SOFA score among patients hospitalized with COVID-19
Source: PLoS One. 2021 Sep 17;16(9):e0257608. doi: 10.1371/journal.pone.0257608 (PMC8448580; doi:10.1371/journal.pone.0257608)
Supplement: Attachment — Submitted filename: PLOS review.pdf [file pone.0257608.s004.pdf]

I wish to congratulate with the Authors for the deep revision of the paper.

I am grateful to PLOS One for the opportunity to review this important study. The revision provided matches all comments and I believe it fits the high-quality standards of the journal.
My name can be public.

Andrea Rossi Zadra

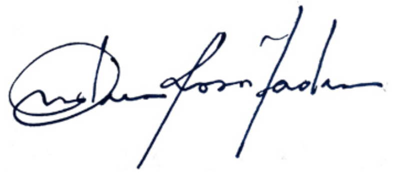

Heart Centre
King Faisal Specialist Hospital
Riyadh
Saudi Arabia
