# Supplementary material for: Racial disparities in the SOFA score among patients hospitalized with COVID-19
Source: PLoS One. 2021 Sep 17;16(9):e0257608. doi: 10.1371/journal.pone.0257608 (PMC8448580; doi:10.1371/journal.pone.0257608)
Supplement: Attachment — Submitted filename: Reviewers comment for PONE-D-21-10700.docx [file pone.0257608.s001.docx]

The author is to be congratulated for submitting this manuscript. The following are the comments:

1. In the current COVID-19 pandemic, this manuscript is ever so important for some countries in which certain residents are more seriously prone to contracting COVID-19. Also, certain ethnic groups have seen both certain morbidity and mortality disparities.
2. In this manuscript, the author reported that non-Hispanic blacks in the United States have significantly higher SOFA scores at upon hospital arrival. Thus, possibly resulting in lesser opportunities to receive critical life-saving medical resources. Yet, the author did not answer the reasons for this phenomenon.
3. One of this manuscript’s major concerns was to evaluate the relationship between ethnicity and systemic racism in the clinical scope. Yet, the author did not separate the Hispanic Americans into Hispanic-Black and other ethnic groups. Thus, causing potential challenges in evaluating the impacts of different skin complexions in certain racism-related issues.
4. The SOFA score is a validated tool to identify patients who have lesser survival opportunities. SOFA should only be used in certain extreme critical situations such as disaster-related ones. Per the author's suggestion, a bonus or prioritization in assessment might be given to certain marginalized populations. This revision of prioritizing triage protocols can only be used in the short term. In the future, the correction of potential systemic racism factors in the clinical scope should be achieved. Detailed discussions about the pro and cons of any protocol revisions or policy change to achieve equality should thus be provided.
5. A stratified study per different ethnic groups is extremely important in medical racism studies. Thus, the supplementary S1 Table “Characteristics of COVID+ patients by Race/Ethnicity” should be moved to the results section. Although the results might not be significant, the data in each cell can provide important information for the readers. A detailed discussion of this table is recommended.
6. Please provide the demographic data of the population serviced by the YNHH, especially those involving the ethnicity, occupation, and other socioeconomic status-related factors.
