# Supplementary material for: Racial disparities in the SOFA score among patients hospitalized with COVID-19
Source: PLoS One. 2021 Sep 17;16(9):e0257608. doi: 10.1371/journal.pone.0257608 (PMC8448580; doi:10.1371/journal.pone.0257608)
Supplement: Attachment — Submitted filename: revision notes 7.26.21.docx [file pone.0257608.s003.docx]

We are deeply grateful for the detailed and thoughtful feedback we received from the editors and from the reviewers. We have endeavored to make all requested revisions within our ability, and to address each comment in the reviewer’s detailed feedback. We hope and believe that the manuscript is significantly improved as a result. Please find comments and our responses below. Please see the revised text for additional revisions that were made to comments attached to the text itself.

From the editors:

1. Please ensure that your manuscript meets PLOS ONE's style requirements, including those for file naming. The PLOSONE style templates can be found at

<https://journals.plos.org/plosone/s/file?id=wjVg/PLOSOne_formatting_sample_main_body.pdf> and

[https://journals.plos.org/plosone/s/file?id=ba62/PLOSOne_formatting_sample_title_authors_affiliations.pdf](about:blank)

We believe we have matched the PLOS ONE style requirements but are happy to make any requested stylistic changes that we have overlooked.

2. Please include your statement regarding patient consent in the manuscript Methods.

 We have modified the following language in study design and data source methods section (page 6):

“The study was approved by the Yale University Human Subjects Committee (study number 2000028081), which judged that the study was exempt from the requirement for consent because data were analyzed anonymously.”

3.We note that you have indicated that data from this study are available upon request. PLOS only allows data to be available upon request if there are legal or ethical restrictions on sharing data publicly. For information on unacceptable data access restrictions, please see <http://journals.plos.org/plosone/s/data-availability#loc-unacceptable-data-access-restrictions>.

We have discussed the sharing of our data with the Yale University Privacy Office which made the determination that we are legally and ethically restricted from sharing data because the extent of data poses a risk of re-identification of patients and their HIPAA protected data through deductive disclosure. Susan Bouregy, PhD ([susan.buregy@yale.edu](mailto:susan.buregy@yale.edu)) is Yale’s chief privacy officer, has a provided a letter documenting this determination that we will upload with our manuscript, and will serve as the contact for the Yale University Privacy Office, to which data requests may be sent.

In your revised cover letter, please address the following prompts:

a) If there are ethical or legal restrictions on sharing a de-identified data set, please explain them in detail (e.g., data contain potentially identifying or sensitive patient information) and who has imposed them (e.g., an ethics committee). Please also provide contact information for a data access committee, ethics committee, or other institutional body to which data requests may be sent.

 Please see prior response.

b) If there are no restrictions, please upload the minimal anonymized data set necessary to replicate your study findings as either Supporting Information files or to a stable, public repository and provide us with the relevant URLs, DOIs, or accession numbers. Please see <http://www.bmj.com/content/340/bmj.c181.long> for guidelines on how to de-identify and prepare clinical data for publication. For a list of acceptable repositories, please see <http://journals.plos.org/plosone/s/data-availability#loc-recommended-repositories>.

Reviewer #1 comments:

 The author is to be congratulated for submitting this manuscript. The following are the comments:
1. In the current COVID-19 pandemic, this manuscript is ever so important for some countries in which certain residents are more seriously prone to contracting COVID-19. Also, certain ethnic groups have seen both certain morbidity and mortality disparities.

We appreciate the reviewer’s enthusiasm for what we believe to be an important topic.

2. In this manuscript, the author reported that non-Hispanic blacks in the United States have significantly higher SOFA scores at upon hospital arrival. Thus, possibly resulting in lesser opportunities to receive critical life-saving medical resources. Yet, the author did not answer the reasons for this phenomenon.

Thank you for emphasizing the importance of an etiological explanation. We have offered several possible explanations (SOFA overestimates mortality among Black patients with COVID, COVID affects Black patients more severely than others, Black patients present/are admitted to hospital later and with more advanced illness) (discussion, page 16). We have expanded on this, suggesting that of these possible explanations anti-Black stigma resulting in distrust of the healthcare system and delayed presentation may be the etiology that fits best with our data and with existing literature, adding the following paragraph on pages 16-17:

“Of these potential contributing factors, prior or current anti-Black discrimination, leading to distrust of the healthcare system and delays in hospital admission, could explain our current findings. Of note, Non-Hispanic Black patients but neither Hispanic patients, patients with Medicaid, nor uninsured patients demonstrated a greater risk of an elevated SOFA score within 24-hours of admission. This would suggest an etiology, such as anti-Black stigma and resulting delays in hospital admission, that affects Black patients in the US to a greater degree than other marginalized populations such as patients with Medicaid or without insurance, who would also be expected to have reduced income, access to preventative care, and other social determinants of health. This potential mechanism accords with prior reports of Black patients in the US presenting to medical attention at more advanced stages of illness.(39, 42) This proposed mechanism is tentative because our analysis does not directly examine income or wealth but uses the imperfect proxy of Medicaid status. Further study, involving more detailed socioeconomic data will be necessary to support or refute this proposed mechanism.”

3. One of this manuscript’s major concerns was to evaluate the relationship between ethnicity and systemic racism in the clinical scope. Yet, the author did not separate the Hispanic Americans into Hispanic-Black and other ethnic groups. Thus, causing potential challenges in evaluating the impacts of different skin complexions in certain racism-related issues.

This is an excellent suggestion, but unfortunately the majority of patients identified as Hispanic ethnicity in the electronic medical record were not also identified by race, so the numbers of patients actually categorized as Hispanic white vs. Hispanic black were too small to analyze. We highlighted this point in the limitation (pages 18-19):

“Our study is also limited by the data available within the clinical EMR. For example, racial and ethnic data is generally documented by unit clerks based on their observation of patients rather than on patient’s self-identification. Patients classified as Hispanic often lack race data, which prevents us from identifying Hispanic Black vs. Hispanic White patients in our analyses.”

4. The SOFA score is a validated tool to identify patients who have lesser survival opportunities. SOFA should only be used in certain extreme critical situations such as disaster-related ones. Per the author's suggestion, a bonus or prioritization in assessment might be given to certain marginalized populations. This revision of prioritizing triage protocols can only be used in the short term. In the future, the correction of potential systemic racism factors in the clinical scope should be achieved. Detailed discussions about the pro and cons of any protocol revisions or policy change to achieve equality should thus be provided.

Thank you for this important suggestion. We agree that adjustments to crisis standards of care will only produce narrow and short-term reductions in health disparities (affecting only a small segment of patients and only during the course of a pandemic), and that broad societal interventions are necessary to reduce health disparities on a systemic and lasting scale. We have added the following text following the description of society-wide interventions (page 18):

“Such interventions, while requiring large investments on a societal level, could reduce health disparities on a much broader and more lasting scale than interventions tailored specifically to the COVID-19 or other pandemics.”

We have added the following text preceding the description of modifications to crisis standards of care (page 18):

Such efforts would not be expected to reduce disparities beyond the setting of a pandemic, and then only for those patients who were subjected to rationing.”

We recommend 8 societal interventions (universal health insurance, a redistributive tax policy, housing support, elimination of food deserts and neighborhood segregation, anti-racism trainings for clinicians, robust collection of race and ethnicity health data, expanded access to higher education, and recruitment of marginalized populations into the medical workforce. While we thought that it was beyond the scope of this manuscript to undertake a detailed discussion of the pros and cons of such major policy interventions, we added new citations to 7 reviews discussing the proposed policies so that interested readers can easily obtain more information. (pages 17-18). We also include a citation discussing the relative advantages and disadvantages of various potential modifications to triage protocols.

5. A stratified study per different ethnic groups is extremely important in medical racism studies. Thus, the supplementary S1 Table “Characteristics of COVID+ patients by Race/Ethnicity” should be moved to the results section. Although the results might not be significant, the data in each cell can provide important information for the readers. A detailed discussion of this table is recommended.

As recommended, we have moved the supplementary S1 table “Characteristics of COVID+ patients by Race/Ethnicity to the results section as table 2. We added the following summary of the table: “Non-Hispanic White patients were significantly older than average while Hispanic patients were significantly younger. Non-Hispanic Black patients had higher rates of elevated BMI and most comorbid conditions, including chronic pulmonary disease, diabetes, hypertension, and advanced renal disease.” Pages 9, 11

6. Please provide the demographic data of the population serviced by the YNHH, especially those involving the ethnicity, occupation, and other socioeconomic status-related factors.

We have added demographic data regarding the population serviced by YNHH, including race, ethnicity, and insurance status. Unfortunately we do not have access to occupation or other socioeconomic data for our patient population (this lacuna is separately identified as a limitation and subject for future research). We state “Across the system, patients are 61% White, 17% Black, 3% Asian, 0.3% Native American, 0.3% Hawaiian or other Pacific Islander, and 19% other or unknown race. Patients are 78% Non-Hispanic, 20% Hispanic, and 2% other or unknown ethnicity. Forty-six percent of patients have private insurance, 19% have Medicare, 28% have Medicaid, and 7% are uninsured.” (Pages 5-6)

In addition, we highlight the need for further study including more detailed socioeconomic data in the discussion (page 19).

Reviewer #2:

*Dear Editor and dear Authors,*

I’m grateful for the opportunity to review this paper, which may be informative on a sensitive topic and add relevant evidence to the available literature on discrimination in healthcare.

Plos One has readers from a wide range of disciplines, and the study may capture a broad interest. For this reason, I believe that some information about the SOFA score, triaging, and the methodology adopted should be discussed further in-depth. Readers who may not be familiar with the topic may perceive an incomplete picture of using such a scoring system -especially for COVID-19 patients, how and where it has been adopted for triaging, and the reasons why it may capture signals of discrimination towards specific populations.

I outlined my comments in the text. More generally, I believe that the concepts in the following sections should be revised.

Thank you for your detailed and thoughtful review. We have attempted to address each of the reviewers comments in the text, as well as below.

Abstract

The research question on disproportionately high SOFA values in Non-Hispanic black patients may refer to a baseline expected value. SOFA is a simple score that does not consider ethnicity or race in the computation, and reasons for potentially elevated values in some populations must rely on other causes.

In background or discussion, we could discuss expected baseline differences as one potential explanation for disparities. With citations. Briefly in intro. Like a sentence saying “another reason SOFA might be discriminatory is because of elevated baseline cre in black people. More in discussion

Thank you for this important suggestion. We have expanded our discussion of possible explanations for the observed disparities in the discussion section (page 16):

“The racial disparities in SOFA scores we found among patients with COVID might be due to the systemic overestimation of mortality among Black persons or underestimation of mortality among White persons. This might occur for example because Black patients at baseline have higher creatinine levels than White patients, leading to an elevation of the renal component of the SOFA score unrelated to any illness.”

We have also added the following citation: Peralta CA, Risch N, Lin F, Shlipak MG, Reiner A, Ziv E, et al. The association of African ancestry and elevated creatinine in the Coronary Artery Risk Development in Young Adults (CARDIA) Study. Am J Nephrol. 2010;31(3):202-8.

Introduction

The use of SOFA score in triaging protocols during the COVID-19 pandemic relies on previous inclusion for disaster medicine protocols in the US. It should be discussed that SOFA has been validated for sepsis and ARDS patients only, it has a predictive value for mortality, but it is not intended -nor it is commonly used- as a tool for the guidance of clinical decisions. Its inclusion in triage protocols has been likely necessary to obtain objective figures to guide ethical decisions rather than clinical approaches. The validity of SOFA in COVID-19 patients is nevertheless questionable because evidence of its predictive performance in such patients would be poor: prognoses of ICU patients with several diseases other than sepsis and ARDS may differ significantly from the expected mortality risk predicted by SOFA. This concern should be addressed on top of its validity for specific sub-populations to deliver a complete picture to readers also from other disciplines. Moreover, the reader may believe that the SOFA score for triaging COVID-19 patients, and rationing resources, is a universal practice, but its actual implementation occurred in some states of the US, and not in Europe.

Thank you for highlighting the need to further describe the limited use of the SOFA score during the pandemic, the fact that the SOFA score was used primarily in the US, that many US crisis standards of care (CSC) incorporated other criteria for triage in combination with the SOFA score, and that US CSC relying on the SOFA score were widely adopted (by States and healthcare systems) but generally not actually activated. We have attempted to do this as suggested in the introduction.

On page 3 we added the following text:

“These policies and recommendations were quickly criticized within their home countries and revised to promote equal access to care, but nonetheless informed the development of early COVID-19 crisis standards of care (CSC) in the United States (US).“

With citation:

4.	Società Italiana Anestesia Analgesia Rianimazione e Terapia Intensiva. DECISIONI PER LE CURE INTENSIVE IN CASO DI SPROPORZIONE TRA NECESSITÀ ASSISTENZIALI E RISORSE DISPONIBILI IN CORSO DI PANDEMIA DA COVID-19 January 13th, 2021. Available from: <https://snlg.iss.it/wp-content/uploads/2021/01/2021_01_13__LINEE-GUIDA_DECISIONI-CURE-INTENSIVE_Def.pdf>.

On page 3, we modified the text to read:

“Most publicly available US triage protocols, prior to and during the pandemic, used the Sequential Organ Failure Assessment (SOFA) score, with or without additional prognostic factors, to assess patients’ likelihood of benefiting (surviving) as a result of receiving medical resources. (9, 11) The rationale was that if a severe shortage of critical medical resources did occur, then the limited resources would be directed to those most likely to survive as a result of receiving them, thereby saving the most lives possible.”

On page 4, we added the following text:

“Triage protocols utilizing the SOFA score were not widespread in Europe and even in the US these CSC were generally not actually activated during the pandemic. Nonetheless, most published US disaster triage protocols prioritized patients who require medical resources but have lower SOFA scores to receive resources, on the grounds that such patients are more likely to benefit (survive), and there is concern that these protocols will guide medical decision making in the US in the event of future public health emergencies.”

Thank you also for highlighting the lack of prior validation of the SOFA score in the context of COVID-19, and the more recent data from the pandemic that have showed the poor prognostic accuracy of COVID in comparison to the APACHE II score or even chronologic age.

We have added the following text to the introduction (page 4):

“Originally developed and validated among septic patients in the medical ICU, subsequent research during the COVID pandemic has shown that the SOFA score is actually poorly predictive of mortality among patients with acute respiratory distress syndrome (ARDS) in the setting of COVID-19 infection; it is less accurate than either the Acute Physiology and Chronic Health Evaluation (APACHE) II score or chronologic age.” (14, 15)

With the following citations:

14.	Zou X, Li S, Fang M, Hu M, Bian Y, Ling J, et al. Acute Physiology and Chronic Health Evaluation II Score as a Predictor of Hospital Mortality in Patients of Coronavirus Disease 2019. Crit Care Med. 2020.

15.	Raschke RA, Agarwal S, Rangan P, Heise CW, Curry SC. Discriminant accuracy of the SOFA score for determining the probable mortality of patients with COVID-19 pneumonia requiring mechanical ventilation. JAMA. 2021;325(14):1469-70.

Methods

The assumption that mean SOFA values may differ across subpopulations of patients, such differences are likely due to social inequalities leading to inequities in healthcare, rather than to hospital- and patient-level features. Acknowledge this point. Anchor disparities in known demonstrated disparities.

Thank you for emphasizing the likely causal relationship between inequalities in social determinants of health and resulting health inequities. We have tried to emphasize this causal pathway closely following the reviewer’s recommended language (pages 4-5).

“These pervasive inequalities in social determinants of health, and the health inequities that they cause, constitute a structure of systemic racism and contribute to higher rates of COVID-19 infection, more severe acute illness due to preexisting conditions, and higher mortality rates. (25, 26)

Given that marginalized populations are more likely to become sicker with COVID-19, utilization of CSC triage protocols, which rely on the SOFA score, have the potential to disproportionately deny medical resources to racial and ethnic minorities. (27, 28) Racial, ethnic, and socioeconomic inequities in health outcomes are a consequence of inequalities in social determinants of health, and they can potentially be exacerbated by triage protocols, as documented in patient cohorts with sepsis and ARDS but not previously examined in patients with COVID-19. (29) The use of SOFA as a criterion to allocate scarce medical resources has the potential to exacerbate inequities caused by social determinants of health.”

With citations:

25.	Egede LE, Walker RJ. Structural Racism, Social Risk Factors, and Covid-19—A Dangerous Convergence for Black Americans. N Engl J Med. 2020.

26.	Garcia MA, Homan PA, García C, Brown TH. The Color of COVID-19: Structural Racism and the Pandemic’s Disproportionate Impact on Older Racial and Ethnic Minorities. The Journals of Gerontology: Series B. 2020.

27.	Cleveland Manchanda E, Couillard C, Sivashanker K. Inequity in Crisis Standards of Care. N Engl J Med. 2020.

28.	White DB, Lo B. Mitigating inequities and saving lives with ICU triage during the COVID-19 pandemic. Am J Respir Crit Care Med. 2021;203(3):287-95.

29.	Ashana DC, Anesi GL, Liu VX, Escobar GJ, Chesley C, Eneanya ND, et al. Equitably Allocating Resources During Crises: Racial Differences in Mortality Prediction Models. Am J Respir Crit Care Med. 2021.

Institutional features of local Medicaid implementation should be described to better deliver what patients are eligible by income thresholds and if there are copayments/deductibles and for which healthcare services.

Thank you for this recommendation. We have added the following text and citations to the methods section describing local Medicaid implementation (page 6):

“Connecticut and Rhode Island Medicaid cover individuals who are citizens, US nationals, permanent residents, or legal aliens with low income. (30, 31) Emergency Medicaid, available to all residents regardless of immigration status, during the COVID-19 public health emergency was expanded to include COVID-19 treatment and hospitalization. (32, 33) Personal asset and income limits vary across states and within states and are available. (30, 31, 34) Generally, Medicaid enrollees in Connecticut and Rhode Island do not pay premiums, deductibles, or copayments.”

With citations:

30.	Benefits.gov. Connecticut Medicaid 2021 [Available from: <https://www.benefits.gov/benefit/1622>.

31.	Benefits.gov. Rhode Island Medical Assistance (Medicaid) 2021 [Available from: <https://www.benefits.gov/benefit/1639>.

32.	The Office of Governor Ned Lamont. Governor Lamont Provides Update on Connecticut’s Coronavirus Response Efforts 2020 [Available from: <https://portal.ct.gov/Office-of-the-Governor/News/Press-Releases/2020/04-2020/Governor-Lamont-Coronavirus-Update-April-30>.

33.	Rhode Island Department of Health. COVID-19 Information: Resources for Undocumented Immigrants 2020 [Available from: <https://covid.ri.gov/public/undocumented-immigrants>.

34.	Brooks T, Roygardner L, Artiga S, Pham O, Dolan R. Medicaid and CHIP eligibility, enrollment, and cost sharing policies as of January 2020: Findings from a 50-state survey 2020 [Available from: <https://www.kff.org/medicaid/report/medicaid-and-chip-eligibility-enrollment-and-cost-sharing-policies-as-of-january-2020-findings-from-a-50-state-survey/>.

Home care should be included as a variable to capture old fragile patients also covered by Medicaid. Disability should also be included as a variable to capture fragile young patients included in Medicare.

Income is an established risk factor for poor health, and its role in affecting severity at admission should be explored. Enrolment in Medicaid discriminates the eligibility threshold but does not provide information for health inequalities in low-middle income classes.

Thank you for the excellent suggestion to include income class among predictor variables. Unfortunately we do not have access to this information for our patients. Income class is not typically captured as operational data in EHRs in the US.

We acknowledge that Medicaid is a poor proxy for low socioeconomic status. Although we do not have accurate or complete data on disability or home care within the electronic medical record, we did examine the number of patients on Medicare <65 (who are presumably enrolled due to disability) – there were 159 such patients and re-running the analysis with these patients excluded did not change the results.

We now emphasize in the discussion the limitations of this approach and recommend further study with more direct and comprehensive measurement of socioeconomic and disability status.

We have identified these lacunae, in the limitation section within our discussion (page 19):

“In particular, our EMR does not include socioeconomic data beyond insurance status, which is a poor surrogate for income, wealth, education, or occupation. Our insurance data are further limited because home care and disability data are not available. Evaluating the degree to which racial and ethnic disparities in the US, such as those identified in this study, are caused by or independent of socioeconomic inequality is a critical question that will require future study with detailed socioeconomic data.”

With citation:

53.	Nuru-Jeter AM, Michaels EK, Thomas MD, Reeves AN, Thorpe Jr RJ, LaVeist TA. Relative roles of race versus socioeconomic position in studies of health inequalities: a matter of interpretation. Annu Rev Public Health. 2018;39:169-88.

I believe that introducing the SOFA as a dichotomic outcome variable in the proposed model may be a severely questionable point. The reader not used to critical care scores may not be able to fully discriminate the risk of mortality associated with the SOFA score. The threshold of 6 points introduces a threshold between a very low risk of mortality (10%) and a low, a high and a very high risk. The use of the 6-point threshold to allocate ICU resources has been implemented in very few jurisdictions, and other criteria were adopted together with SOFA, while most institutions and government directives use h thresholds higher than 6. The outcome SOFA variable should be changed into class variables accounting for mortality risk: this may increase the sensitivity of the analysis in capturing specific subpopulations at high risk of mortality and their relative risk compared to other subpopulations, e.g. ethnic and race groups or income classes.

Thank you for raising the legitimate concern about dichotomization of the SOFA score. We focused our analysis at the cut-off of 6 because most US crisis standards of care (including the New York State protocol referenced in Wunsch’s comparison) defined a group that was most prioritized to receive critical care resources on the basis of a low SOFA score (in the case of the New York State protocol this group was defined by a SOFA score of 6 or less). (https://nysba.org/app/uploads/2020/05/2015-ventilator_guidelines-NYS-Task-Force-Life-and-Law.pdf; Manchanda et al. Crisis Standards of Care in the USA. Journal of Racial and Ethnic Disparities). While the New York State Protocol and other US protocols defined other categories that were less prioritized or even forbidden from receiving ICU resources (eg in the New York State protocol those with SOFA score >11), this high-SOFA group was extremely small (2% of patients in our cohort, as compared to 14% of patients with SOFA 6 or higher). We were unable to conduct an analysis that further subdivided patients with elevated SOFA scores (6 or higher) into narrower categories, because of the very small size of the high-SOFA groups.. We have now emphasized this point within our limitations (page 19):

“Our study is also limited by the relatively small number of patients with very elevated SOFA scores, which prevented analysis of SOFA scores as a categorical rather than dichotomous outcome variable.”

Given the specific research question, the interaction between Non-Hispanic Black and income should be explored; alternatively, the interaction between Non-Hispanic Black patients and Medicaid plan should be introduced.

Thank you for this excellent suggestion. While we do not have access to income data, we did examine the interaction between Non-Hispanic Black patients and Medicaid. There was no significant interaction (p-value of 0.1294).

Moreover, the differences by subpopulations and their likely urban concentration strongly suggest adding upon a hospital-level stratification that can be more informative than a patient-level alone, and the available data allows this stratification by hospitals. Then, a multilevel model capturing hospital-level differences is strongly recommended.

If the finding by ethnic/race groups is confirmed, very robust evidence will be provided for potential discrimination due to social factors and systemic racism. Ask Deron. If not doable, we will need to articulate reason why not feasible.

Thank you for highlighting the potential importance of hospital-level differences. As recommended, we conducted a multiple mixed-effects logistic regression, including random intercepts placed on hospital of admission to account for potential inter-hospital variability, with unchanged results. Please see the essentially identical results below:

|  | **Not accounting for clustering of patients within hospitals** | | | | **accounting for clustering of patients within hospitals** | | | |
| --- | --- | --- | --- | --- | --- | --- | --- | --- |
| **Characteristic** | **Model 1- Combined Multivariate** | | | | **Model 1- Combined Multivariate** | | | |
|  | **OR** | **95% CI** | | **p-value** | **OR** | **95% CI** | | **p-value** |
| **Race/Ethnicity** |  |  |  |  |  |  |  |  |
| Hispanic | 0.87 | 0.62 | 1.24 | 0.4501 | 0.87 | 0.61 | 1.24 | 0.4468 |
| Black Non-Hispanic | 1.49 | 1.11 | 1.99 | 0.0075 | 1.49 | 1.11 | 1.99 | 0.0078 |
| White Non-Hispanic | *reference* | | | | *reference* | | | |
| **Age** |  |  |  |  |  |  |  |  |
| 18-34 | *reference* | | | | *reference* | | | |
| 35-64 | 1.95 | 1.07 | 3.54 | 0.0288 | 1.95 | 1.07 | 3.54 | 0.0287 |
| >=65 | 2.57 | 1.32 | 4.98 | 0.0052 | 2.57 | 1.32 | 4.97 | 0.0053 |
| **Sex** |  |  |  |  |  |  |  |  |
| Men | 1.94 | 1.51 | 2.50 | <.0001 | 1.94 | 1.51 | 2.50 | <.0001 |
| Women | *reference* | | | | *reference* | | | |
| **Insurance status** |  |  |  |  |  |  |  |  |
| Private | *reference* | | | | *reference* | | | |
| Medicare | 1.07 | 0.70 | 1.63 | 0.757 | 1.07 | 0.70 | 1.62 | 0.7659 |
| Medicaid | 1.35 | 0.89 | 2.04 | 0.1536 | 1.35 | 0.89 | 2.03 | 0.1569 |
| Uninsured | 1.19 | 0.66 | 2.13 | 0.566 | 1.18 | 0.66 | 2.13 | 0.5711 |
| **BMI** |  |  |  |  |  |  |  |  |
| <25 | *reference* | | | | *reference* | | | |
| 25 - 29.9 | 1.22 | 0.88 | 1.68 | 0.2317 | 1.22 | 0.88 | 1.68 | 0.2323 |
| 30-34.9 | 1.30 | 0.91 | 1.87 | 0.1561 | 1.30 | 0.91 | 1.87 | 0.153 |
| 35+ | 1.52 | 1.07 | 2.18 | 0.0207 | 1.52 | 1.07 | 2.18 | 0.0209 |
| **Comorbid conditions** |  |  |  |  |  |  |  |  |
| Chronic pulmonary disease | 1.07 | 0.81 | 1.40 | 0.6453 | 1.07 | 0.81 | 1.40 | 0.6471 |
| CHF | 1.19 | 0.86 | 1.63 | 0.288 | 1.19 | 0.86 | 1.63 | 0.2884 |
| Diabetes | 1.07 | 0.82 | 1.41 | 0.6082 | 1.07 | 0.82 | 1.40 | 0.6128 |
| CAD | 1.18 | 0.86 | 1.61 | 0.3151 | 1.18 | 0.86 | 1.61 | 0.3168 |
| Hypertension | 0.95 | 0.67 | 1.34 | 0.7594 | 0.95 | 0.67 | 1.35 | 0.769 |
| Advance renal disease | 2.35 | 1.62 | 3.40 | <.0001 | 2.35 | 1.62 | 3.40 | <.0001 |
| Advance liver disease | 2.51 | 1.29 | 4.89 | 0.0068 | 2.51 | 1.29 | 4.89 | 0.0069 |

The lack of difference may be in part because during the pandemic, the healthcare system worked as a unified network, with sicker patients transferred from smaller hospitals within the network to Yale New Haven Hospital, all operating under a single protocol.

We added comments on the multiple mixed-effects logistic regression and the unchanged results in the statistical analysis methods section (page 9), and the results section (page 14).

Discussion

The reason for inequalities in accessing healthcare should be addressed according to the new findings.

We have modified the discussion to highlight potential causes of the identified disparity in initial SOFA scores, and to offer our thoughts as to the most likely causes.

“The racial disparities in SOFA scores we found among patients with COVID might be due to the systemic overestimation of mortality among Black persons or underestimation of mortality among White persons. This might occur for example because Black patients at baseline have higher creatinine levels than White patients, leading to an elevation of the renal component of the SOFA score unrelated to any illness. (37) Alternatively, Black persons with COVID-19 might have higher SOFA scores in the hospital because COVID-19 affects them more severely, for example because they are subjected to higher levels of discrimination and stress or because they have less access to long-term preventive care, quality education, economic stability, and other social determinants of health. (23, 24, 38) Finally, Black patients might have higher SOFA scores at the time of admission because they present or are admitted to hospitals only when they are sicker. (39, 40) This could occur because of current or prior discrimination within the healthcare system that might discourage patients from seeking medical attention with mild or moderate symptoms. (41)

Of these potential contributing factors, prior or current anti-Black discrimination, leading to distrust of the healthcare system and delays in hospital admission, might best explain our current findings. Of note, Non-Hispanic Black patients but neither Hispanic patients, patients with Medicaid, nor uninsured patients demonstrated a greater risk of an elevated SOFA score within 24-hours of admission. This would suggest an etiology, such as anti-Black stigma and resulting delays in hospital admission, that affects Black patients in the US to a greater degree than other marginalized populations such as patients with Medicaid or without insurance, who would also be expected to have reduced income, access to preventative care, and other social determinants of health. This potential mechanism accords with prior reports of Black patients in the US presenting to medical attention at more advanced stages of illness.(39, 42) Further study, involving more detailed socioeconomic data will be necessary to support or refute this proposed mechanism.”

I hope my suggestions may be helpful to improve the overall quality of the paper for publishing in Plos One.

My identity can be public.

Best regards,

Andrea Rossi Zadra

Critical care consultant

King Faisal Specialist Hospital and Research Centre

Riyadh

Saudi Arabia

We are deeply grateful for the detailed and thoughtful comments. We have done our best to address each comment (here and in the text of the paper) and are hopeful that the paper is significantly improved as a result.

Please see the revised text for additional revisions that were made to comments attached to the text itself.
