# Supplementary material for: Racial disparities in the SOFA score among patients hospitalized with COVID-19
Source: PLoS One. 2021 Sep 17;16(9):e0257608. doi: 10.1371/journal.pone.0257608 (PMC8448580; doi:10.1371/journal.pone.0257608)
Supplement: Attachment — Submitted filename: revision notes 8.31.21.docx [file pone.0257608.s005.docx]

Again, we are deeply grateful for the detailed and thoughtful feedback we received from the editors and from the reviewers. We have endeavored to make all requested revisions, including the most recent revision request from reviewer 1. We hope and believe that the manuscript is significantly improved as a result. Please find comments and our responses below.

Reviewer #1: Table 2 (Characteristics of COVID+ patients by Race/Ethnicity) showed the vulnerability of Non-Hispanic Black patients in their health and socioeconomic status, which may lead to higher SOFA scores. Since this manuscript intended to explore the racial disparities in SOFA Score, the significance of this table is worth mentioning in the discussion section.

Thank you very much for this important recommendation. We agree that it is important to emphasize that Non-Hispanic Black patients face elevated levels of chronic comorbidities that are associated with elevated SOFA scores, as demonstrated in table 2. At the same time, Non-Hispanic Black patients’ increased risk of an elevated SOFA score persisted even when insurance status and chronic comorbidities were controlled for in the multivariate analysis. We have added the following language in the first paragraph of the discussion, specifically referencing Table 2 and its significance (page 16):

“Non-Hispanic Black patients were more likely to suffer from chronic comorbidities associated with elevated peak SOFA scores such as obesity and advanced renal disease (Table 2), and their risk of an elevated SOFA score persisted even when such comorbidities and other risk factors were controlled for in the multivariate analysis (Table 4). Neither insurance status nor exposure to chronic comorbidities fully explained the increased risk faced by Non-Hispanic Black patients with COVID-19.”

We are deeply grateful for the detailed and thoughtful comments. We have done our best to address each comment (here and in the text of the paper) and are hopeful that the paper is significantly improved as a result.
